# An Adaptive Partial Least-Squares Regression Approach for Classifying Chicken Egg Fertility by Hyperspectral Imaging

**DOI:** 10.3390/s24051485

**Published:** 2024-02-24

**Authors:** Adeyemi O. Adegbenjo, Li Liu, Michael O. Ngadi

**Affiliations:** 1 Department of Bioresource Engineering, McGill University, 21111 Lakeshore Road, Ste-Anne-de-Bellevue, Montreal, QC H9X 3V9, Canada; li.liu5@mcgill.ca; 2 Department of Agricultural and Environmental Engineering, Obafemi Awolowo University, Ile-Ife 220005, Nigeria

**Keywords:** chicken egg fertility, classification, PLS regression, hyperspectral imaging

## Abstract

Partial least-squares (PLS) regression is a well known chemometric method used for predictive modelling, especially in the presence of many variables. Although PLS was not initially developed as a technique for classification tasks, scientists have reportedly used this approach successfully for discrimination purposes. Whereas some non-supervised learning approaches, including, but not limited to, PCA and k-means clustering, do well in identifying/understanding grouping and clustering patterns in multidimensional data, they are limited when the end target is discrimination, making PLS a preferable alternative. Hyperspectral imaging data on a total of 672 fertilized chicken eggs, consisting of 336 white eggs and 336 brown eggs, were used in this study. Hyperspectral images in the NIR region of the 900–1700 nm wavelength range were captured prior to incubation on day 0 and on days 1–4 after incubation. Eggs were candled on incubation day 5 and broken out on day 10 to confirm fertility. While a total number of 312 and 314 eggs were found to be fertile in the brown and white egg batches, respectively, the total number of non-fertile eggs in the same set of batches was 23 and 21, respectively. Spectral information was extracted from a segmented region of interest (ROI) of each hyperspectral image and spectral transmission characteristics were obtained by averaging the spectral information. A moving-thresholding technique was implemented for discrimination based on PLS regression results on the calibration set. With true positive rates (TPRs) of up to 100% obtained at selected threshold values of between 0.50 and 0.85 and on different days of incubation, the results indicate that the proposed PLS technique can accurately discriminate between fertile and non-fertile eggs. The adaptive PLS approach was, thereby, presented as suitable for handling hyperspectral imaging-based chicken egg fertility data.

## 1. Introduction

Out of 13.1 billion hatching eggs produced by the U.S. egg industry for the year 2005, the ratio of layer to broiler eggs produced was reported to be around 12:1, creating different degrees of discriminating tasks to both layer and broiler industries. With fertility rates in the range of 60 to 90% [1], there could be about 1.3 billion to over 5 billion infertile eggs being incubated yearly. According to the 2013 Agriculture and Agri-Food Canada report, the total hatching egg product was set at 798.3 million, resulting in a minimum of about 80 million non-fertile eggs being incubated annually in Canada alone, which is worth a whopping sum of about $27.6 million being lost annually. Furthermore, the discarding of non-hatching eggs has consistently posed significant disposal problems for hatcheries, especially in the case of exploder eggs in hatching cabinets, resulting in a high tendency of molds and bacterial infestations in other eggs [1]. Thus, the identification and isolation of infertile eggs from fertile eggs have significant economic and safety implications for commercial broiler breeders.

Indeed, research efforts have supported the great potential applications of hyperspectral imaging as a non-destructive method for assessing fertility/hatchability, embryo development, and mortality rates in chicken eggs. These studies have, however, reported mostly on the fertility detection of white-shelled chicken eggs, with scanty reports on brown eggs and, where available, results were not as promising as with white eggs. Also, samples considered in earlier studies were small [1,2,3]. Ref. [2] reported low validation and verification accuracies for fertility detection in brown eggs (validation data sets: 71% for day 0; 63% for day 1, 65% for day 2, 83% for day 3; verification data sets: 51% for day 0 and 50% for day 3). It was concluded that the Mahalanobis Distance (MD)/Principal Component Analysis (PCA) model used was not adequate for the discrimination. This is of great concern for the poultry industry as this means a large number of fertile eggs would end up being discarded based on such a model. Hence, there is indeed an urgent need for more appropriate discrimination techniques for chicken egg fertility assessments.

Partial least-squares (PLS) regression, also commonly known as the projection to latent structure [4], is a widely used technique that has useful applications in various domains, including, but not limited to, engineering, medicine, and agriculture. The PLS approach is particularly known for building predictive models with many variables, rather than explaining underlying correlations between variables [5]. PLS was not initially developed as an approach for statistical classification tasks, except for regression; nonetheless, scientists have reportedly used this approach successfully for discrimination purposes [6,7]. Such application domains include, but are not limited to, discriminating between Arabica and Robusta coffee beans, classifying soy sauce by geographic region, and in dementia diagnosis research [8,9,10]. Ref. [11] used a PLS-based method for non-destructive fertilization status classification and egg odor characterization. This work, albeit with promising results, did not report any confirmatory fertility status check for reference data and, therefore, did not take into consideration the clear occurrence of the fertility status change during incubation periods. In the same vein, [12] used a PLS-based optimization strategy for pigeon egg fertility identification. This work reported a promising overall accuracy result of 98.6% using 30 latent variables (LVs), in conjunction with a standardized mean centering pre-processing. Apart from the fact that this work was for pigeon eggs and it was worthwhile to test this method on chicken eggs, the number of LVs were quite high, and the results were for day 3 of incubation and reported no outcomes for day 0, prior to incubation. There is, therefore, still a dire need for addressing early egg fertility detection problems before incubation.

Even though principal component analysis (PCA) is a well known chemometric method that has recorded notable success as a pre-classification procedure, this success has been reported to be only possible in various application domains because of its favorable disposition to considering among-group variability rather than within group variability [6]. This mode of PCA implementation, therefore, does not address a situation in which the within-group variability in data is also a major concern due to the existence of several sub-clusters in a single class not having the same number of samples [13]. The within-class variability occurrence has been reported to have unfavorable consequences on learning algorithms [14]. In such a situation, PCA has been observed to perform less optimally, thereby presenting PLS as the next applicable alternative. According to [6], PLS was reported to have the potential to outperform PCA when within-group variability dominated among-group variability. Additionally, PLS has been judged to be versatile in solving data structural problems, like skew distributions, multicollinearity, and missing regressors condition, all which are peculiar characteristics of hyperspectral imaging data [15]. Ref. [3] reported a perfect classification accuracy using PCA and k-means clustering. However, being non-supervised learning techniques, these approaches need further confirmation using standard supervised learning algorithm(s). Although some non-supervised learning approaches, like PCA, k-means, k-medians, and hierarchical cluster analysis, are superb at identifying/understanding grouping and clustering patterns in multidimensional data, they are limited when the end target is discrimination [6]. Furthermore, unsupervised classification is always the starting point in any discrimination problem and should be necessarily followed by supervised classification [16], towards an industrial adoptability consideration. More recent works [17,18,19,20] have attempted approaches, including the state-of-the-art deep learning architecture, for studying chicken egg fertility. Such works, however, were not successful in fertility detection prior to incubation (day 0 of incubation).

In view of the foregoing, this study has, therefore, considered and tested the suitability of an adaptive supervised learning PLS regression approach, together with a threshold-moving technique, in handling chicken egg fertility hyperspectral imaging data. Classification accuracy has been based on the confusion matrix evaluation criterion at the expense of the more general overall accuracy computation, which has been shown to be inappropriate when dealing with data containing a rare class [21], as with non-fertile eggs in chicken egg fertility data.

## 2. Materials and Methods

### 2.1. Samples

A total of 336 brown shell eggs and 336 white shell eggs, with a mean weight of 58–62 g, were received from a commercial fertile egg producer (Simetin Hatchery) in 14 batches (48 eggs per batch) over a period of 3 months. To take advantage of having sizeable minority data in the samples and due to the unavailability of a large enough sample size during the study period, this research adopted the consecutive sampling approach, in which data collected in batches were later analyzed together as a whole when the desired sample size was reached. All eggs used were freshly laid (1–4 days old), preserved at a temperature between 12.8 and 18.30 °C and relative humidity of 75% before and during transportation to the imaging lab [22]. There were 7 batches of eggs collected in each group of brown and white egg sets. Table 1 shows the details of the overall egg samples available for analysis on each day of incubation for both brown and white eggs. Out of the total 336 eggs received for both brown and white eggs, the number of total available eggs eventually used for analysis varied with incubation time due to egg breakage during handling. While 2 eggs (1, day 0; 1, day 1) were broken from the brown egg batch, a total of 3 eggs (1, day 0; 2, day 3) were broken from the white egg batch. This variation in total available eggs during analysis resulted in a slightly different degree of imbalance from one day of incubation to another. The ratio of non-fertile to fertile eggs in this study is estimated from Table 1 to be 1:13 and 1:15 for both brown and white eggs, respectively.

### 2.2. Image Acquisition and Processing

**Figure 1:**
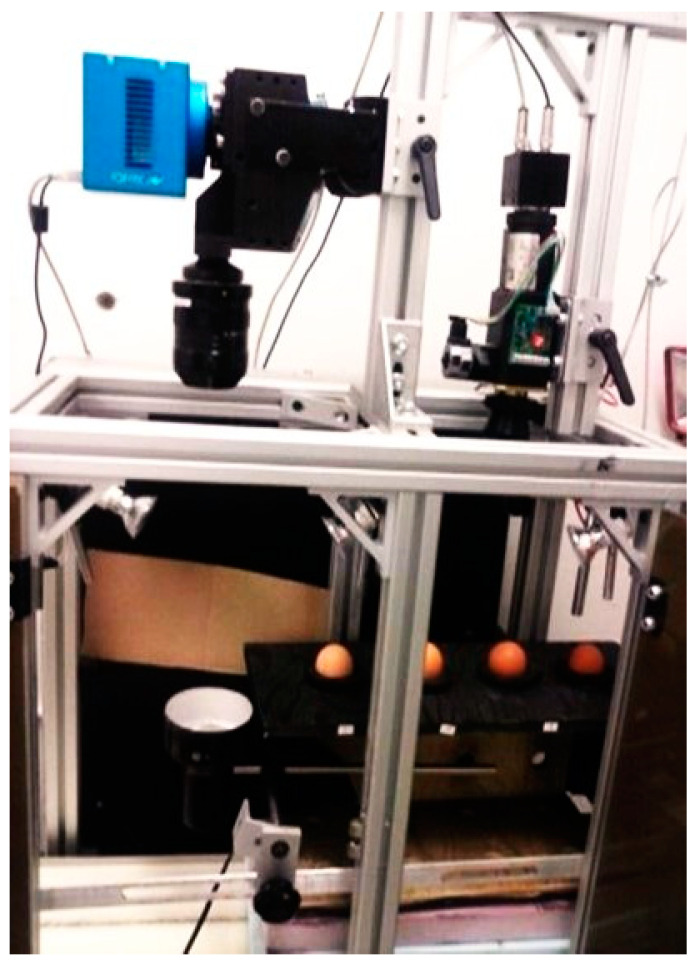
The hyperspectral imaging acquisition system used in the study.

A laboratory near-infrared (NIR) hyperspectral imaging system (Figure 1) used in this project comprised an InGaAs camera; a conveyor (Donner 2200 series, Donner Mfg. Corp., Hartland, WI, USA), driven by a stepping motor (MDIP22314, Intelligent motion system Inc., Marlborough, MA, USA); a line-scan spectrograph (HyperspecTM, Headwall Photonics Inc., Marlborough, CT, USA), with a NIR spectral wavelength range from 900 to 1700 nm and a spectral resolution of 4.79 nm; a tungsten halogen lamp (50 W), providing back illumination to eggs; an enclosure supporting the system; a data acquisition and pre-processing software (“Hyperspec III”, Headwall Photonics Inc., Fitchburg, MA, USA); and a PC. All eggs were first imaged by the hyperspectral imaging system on day 0 (just prior to incubation) and, immediately after imaging, the eggs were incubated in an Ova-Easy 190 Advance Series II Cabinet Incubator (Brinsea Products Inc., Titusville, FL, USA) at 37.78 °C (100 °F) and 55% relative humidity. The eggs were automatically turned every hour. On days 1, 2, 3, and 4 of incubation, the eggs were removed for imaging in sequence and then immediately returned to the incubator, in a process of about 1 min.

After 10 days of incubation, eggs were candled and broken out to determine fertility [23]. The resulting classification is as shown in columns 5 and 6 of Table 1a,b for both brown and white eggs. The output hypercube HSI image obtained is 800 rows × 320 columns × 167 bands. The region of interest (ROI) of individual whole-egg spectral images was selected at a maximum wavelength band 38 (1076 nm) and punched through other wavelength bands. A mask was created for each individual egg to segment it from the original spectral image that normally included four eggs. Segmented individual eggs were then used for calculating mean spectra, following standard procedures.

### 2.3. Spectral Transmission and Feature Extraction

Spectral transmission characteristics, namely mean spectral (MS) and extracted features based on thresholding, were used in this study for further data analysis. MS stands for the mean value of all pixels in ROI for the current wavelength over the spectral range of 900–1700 nm. The threshold-moving method has been used in cost-sensitive neural network learning with reported good effectiveness, even with highly imbalanced data sets. A more detailed explanation of this method is described by [24,25]. Threshold (TR) values considered for extraction of features in the present work ranged between 0.50 and 0.85. The purpose of adopting the thresholding technique in conjunction with a PLS algorithm was to extract useful spectral features to facilitate the discrimination of fertile eggs from non-fertile eggs. With the choice of an appropriate threshold value, a new set of features with the potential of achieving optimal classification accuracy was extracted for analysis, and discrimination performance was then evaluated using the confusion matrix evaluation criterion. All operations were performed on the MATLAB R2014a (The MathWorks, Inc., Natick, MA, USA) platform. To take advantage of having sizeable minority data in the analysis, we used a consecutive sampling data collection approach, in which data collected in batches were analyzed together as a whole as at the time the desired sample size was reached.

### 2.4. Partial Least-Squares Regression Analysis

For the different days of egg incubation, a PLS code written in the MATLAB R2014a environment was used for data analysis and full cross-validation was later employed as a means of internal validation in all cases. Unlike the popular multiple linear regression (MLR), which is prone to the problem of over-fitting in the presence of too many factors, PLS analysis adjusts to the over-fitting problem by extracting only the latent factors accounting for majority of the manifest factor variation [26]. Not only this, PLS is well known for analyzing data with strongly collinear (correlated), noisy, and numerous X-variables. The PLS analysis as adopted in this study modelled both the X- and Y-matrices simultaneously (thereby maximizing the covariance between X and Y) to reveal the latent variables in X, having shown the potential of accurately predicting latent variables in [27]. Unlike the PCA, which decomposes X to obtain components that explain the most variability in X, PLS seeks to identify components from X that best predict Y [28].

#### 2.4.1. Choice of Optimal Number of PLS Components (PCs)

The PLS modelling process is greatly influenced by only few underlying (latent) variables; however, the appropriate number of these latent variables is usually unknown. One major aim of PLS analysis, therefore, was to estimate this number [27] and, in doing so, it became very critical to identify an optimum value for the user- defined number “*n*” of PLS components (PCs), which is directly related to the selection of informative features required for an accurate discrimination process. This study followed the full (leave one out) cross-validation (CV) procedure reported by [27] in testing the predictive performance of PLS components and stopping when the addition of more components tended to reduce performance. A detailed explanation of this procedure has been described elsewhere [29,30,31]. Nevertheless, because the end goal in this study was discrimination and not regression, the traditional interpretation of the CV procedure cannot be applied directly in its entirety and is the reason why the confusion matrix criterion was adopted for evaluating discrimination performance. PCs ranging from *n* = 5 to *n* = 50 (in interval of 5) were tested for classification accuracy before arriving at an optimum value for “*n*”. The threshold for initial feature extraction was chosen to be 0.80 from preliminary trial and error analysis. This threshold was subsequently used in predictive performance testing for determining the optimum number of PCs.

#### 2.4.2. Criteria for Evaluating Discrimination Performance

Overall accuracy has been presented as inappropriate for measuring classifier performance in a situation consisting of non-prevalent class data [21,32]. Unlike in earlier similar studies as reported in [33], where smaller samples were used and evaluations were made based only on overall accuracy, the present study adopted the confusion matrix evaluation criterion for a binary-class egg fertility discrimination problem. This was to be able to see performance clearly under an imbalanced data situation. The problem with class imbalance is that, even though overall classification accuracy can be high, it is always in favor of the prevalent class and, therefore, the final model does not usually generalize in real-time applications. In a binary class classification situation, the specific class with very few training samples but with high identification importance is commonly referred to as the positive class and the other as the negative class [34]. This definition, however, does not seem to be directly applicable to most agricultural and food processing operations. Even though non-fertile eggs in this research belong to the rare class (very few training examples), fertile eggs of the majority class are of higher identification importance from the hatchery industry’s point of view. Therefore, agricultural and food processing applications might not fit in directly to the definition of positive class being the class with very few training samples and simultaneously of higher recognition importance. Nonetheless, in this first study, we have maintained taking non-fertile eggs as the positive class, not only because they fall into the minority class but also because the future industrial instrumentation for egg fertility assessments might be much more economically built and viable to reject non-fertile eggs (fewer samples) and accept fertile eggs (larger number of samples). The choice of our true positive class in this first study is critical to be able to examine our results, while maintaining conventional consistency. The confusion matrix employed for the interpretation of the PLS analysis and, hence, determining classification accuracy is as shown in Table 2, where TPR, FPR, TNR, and FNR represent the rate in percentage of true positive examples, the rate in percentage of false positive examples, the rate in percentage of true negative examples, and the rate in percentage of false negative examples, respectively. If TP = True positive (number of non-fertile eggs classified as non-fertile), TN = True negative (number of fertile eggs classified as fertile), FP = False positive (number of fertile eggs classified as non-fertile), and FN = False negative (number of non-fertile eggs classified as fertile), the following equations as reported in [34,35,36] can be obtained and the traditional overall accuracy (OVA), including the error rate (ERR), can be computed as:

TPR = TP/(TP + FN) × 100(1)

TNR = TN/(TN + FP) × 100(2)

FPR = FP/(FP + TN) × 100(3)

FNR = FN/(FN + TP) × 100(4)

OVA = (TP + TN)/(TP + FN + FP + TN) × 100(5)

## 3. Results and Discussion

**Figure 2:**
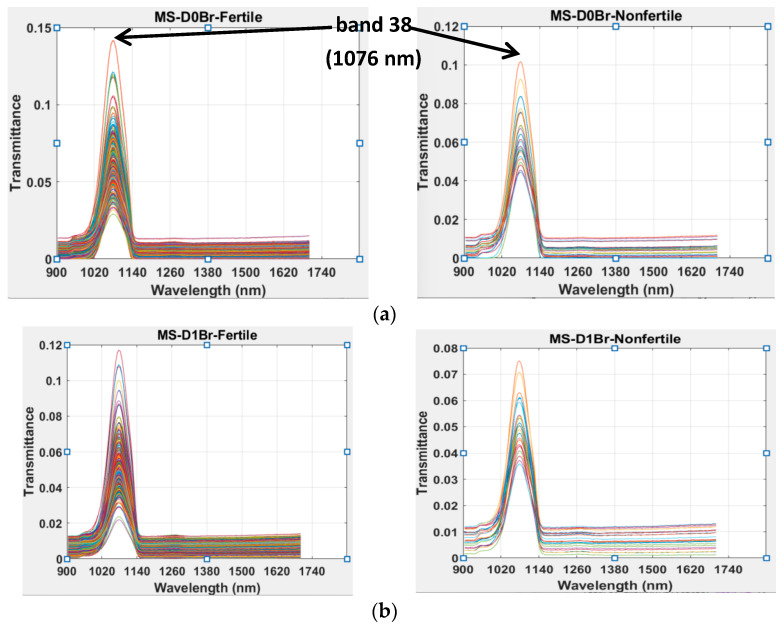

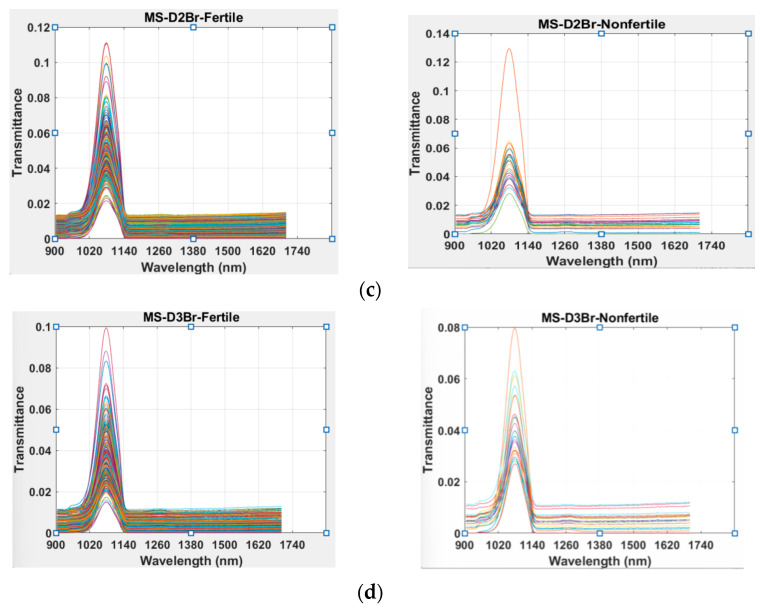
Typical transmittance mean spectra (MS) profiles of brown eggs on different days of incubation (**a**), day 0 (**b**), day 1 (**c**), day 2 (**d**), day 3.

While a total number of 312 and 314 eggs were found to be fertile (F) in the brown and white egg batches, respectively, the total numbers of non-fertile (NF) eggs in the same set of batches were 23 and 21, respectively (see Table 1), at the start of our analysis. Figure 2 showed typical transmittance MS profiles of brown eggs on different days of incubation. A notable peak identified from the transmittance spectrum of both F and NF eggs (Figure 2) was at around band 38 (band regions 20–60), corresponding to the wavelength regions of 987–1177 nm. The absorption peak in this region was observed to be due to the second overtone molecular stretching of the N-H, O-H, and C-H chemical bonds. Since these chemical bonds are present in both F and NF eggs, discrimination at this point was, therefore, based on considering transmittance intensity differences or differences in the concentration of the chemical compounds (protein, water, and fat) responsible for the observed peaks in both egg types [37]. It was further observed from Figure 2 that the fertile eggs maximum transmittance intensity decreased as incubation period increased from day 0 to day 3. This observation might be related to the onset of active molecular activities from meiotic and mitotic cell divisions in the fertile eggs. Knowing that the proportion of light absorbed by any material is dependent on the quantity of molecules involved in molecular interaction, fertile eggs tend to absorb more light at different wavelengths as incubation period progresses and, hence, the amount of light being transmitted decreases accordingly. The non-fertile eggs transmission intensity over the considered incubation periods did not follow a definite trend. The initial decrease in maximum transmittance intensity from day 0 to day 1 might also be related to the molecular interactions from meiotic cellular division. As the meiosis process terminated in the non-fertile eggs, further cell division also ceased since there was no fertilization to trigger the onset of mitotic cell division [33]. Therefore, the subsequent egg maximum intensity increase and later decrease can be attributed to the degree of albumen–yolk solution concentration, which is dependent on the rate of yolk dissolution into the albumen under incubation conditions. From Beer’s law, solution concentration is directly proportional to light’s absorption [38,39] up to a specific level, as the law failed at some higher concentrations.

**Figure 3:**
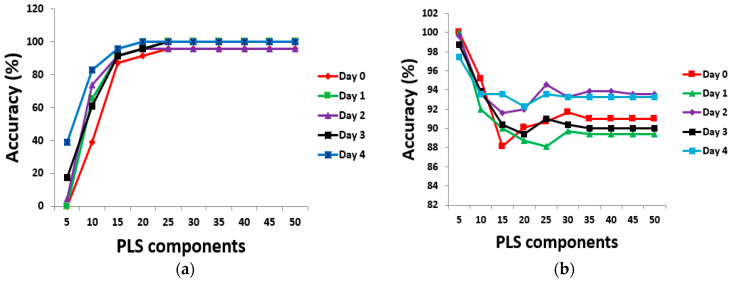
Determining the optimum number of PLS components for brown eggs based on (**a**) TPR and (**b**) TNR.

**Figure 4:**
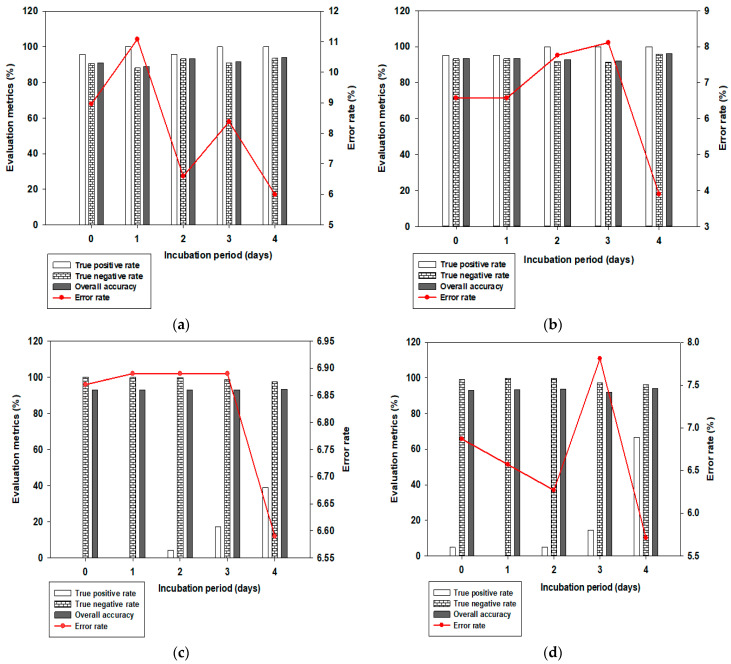
Evaluation metrics (%) for built models on different days of incubation of (**a**) brown eggs, 25 PCs; (**b**) white eggs, 25 PCs; (**c**) brown eggs, 5 PCs; and (**d**) white eggs, 5 PCs.

Using brown egg data, Figure 3 shows the predictive performance chart for determining the optimum number of PLS components for different days of egg incubation. The TPR performance chart (Figure 3a) showed that adding more components above 25 did not bring any further improvement to classification accuracy. Twenty-five PLS components were then chosen for feature extraction and subsequent discrimination based on the TPR performance results. However, if the TNR was of greater or equal interest, only the first 5 PLS components would suffice for further feature extraction (see Figure 3b). In light of the above, further analysis in this study used both 25 and 5 PLS components at various selected thresholds between 0.50 and 0.80 for feature extraction and eventual classification. Figure 4 shows evaluation metrics at a threshold point of 0.80 for both brown and white eggs on different days of incubation, with associated misclassification error rates.

From Figure 4a on day 0 of incubation, brown eggs achieved TPR, TNR, OVA, and ERR of 95.65%, 90.71%, 91.04%, and 8.96, respectively. TPR increased to 100% on day 1 of incubation but at an increased error rate (ERR) of 11.08. For day 2 of incubation, TPR, TNR, OVA, and ERR were 95.65%, 93.25%, 93.41%, and 6.59, respectively. The day 3 incubation model, like the day 1 model, achieved 100% TPR but at an increased ERR of 8.38 compared to day 2 of incubation. From the four evaluation metrics considered, the day 2 incubation model was observed to be more optimized in comparison to others. Even though day 4 of incubation had the best model accuracy parameters of 100%, 93.57%, 94.01%, and 5.99 for TPR, TNR, OVA, and ERR, respectively, it might not be chosen as an appropriate model considering choice criteria of early detection, optimized use of incubation energy, space, and resources. From Figure 4b on day 0 of incubation, white eggs achieved TPR, TNR, OVA, and ERR of 95.24%, 93.31%, 93.43%, and 6.57, respectively. All evaluation metrics for white eggs remained practically the same for day 1 of incubation as for day 0 of incubation, unlike in brown eggs where day 1 model improvement was at a detrimental increase in error rate (ERR). For day 2 of incubation, TPR, TNR, OVA, and ERR for white eggs were 100%, 91.72%, 92.74%, and 7.76, respectively. The day 3 incubation model, just like the day 2 model, also achieved 100% TPR but at an increased ERR of 8.11, 91.35% TNR, and 91.89% OVA. Just like the brown egg set, the day 4 incubation model for white eggs also had the best model accuracy parameters and lowest error rate of 3.90, but might not necessarily be the overall preferred model based on uttermost consideration of the early detection criterion and other conditions previously mentioned. Overall, white egg models performed better than brown egg models and this may not be unconnected with the interference of brown eggshell pigmentation effect on the degree of light transmittance into the intact egg. With this observation, the possibility of building a single multi-purpose model to handle both egg types will need to carefully consider mitigating the effect of eggshell pigmentation on light transmittance. From Figure 4a,b at 25 PCs, both brown and white eggs achieved 100% TPR accuracy on days 3 and 4, while neither of the two sets of eggs achieved 100% TPR accuracy on day 0 of incubation. With TPRs for both types of egg sets being higher than the TNRs, the built models were much better in recognizing non-fertile eggs than fertile eggs. At 5 PCs (Figure 4c,d), neither of the two sets of eggs achieved perfect TPR accuracy of 100% on any incubation day considered. 100% TNR accuracy was, however, obtained on days 0 and 1 for brown eggs, but also at a detrimental 0% TPR corresponding to accuracies. Hence, the classifier, despite classifying all fertile eggs as fertile, will also end up misclassifying all non-fertile eggs as fertile on these 2 days of incubation. The lowest misclassification error rates for the classes of eggs at both 25 and 5 PCs considered were on day 4 of incubation, but this day is already too late for early recognition and classification, leaving us to consider earlier days (especially day 0) more critically. Indeed, we need a classifier model that will perform at a much closer margin of TPR and TNR accuracies and, at the same time, use much fewer PCs.

**Figure 5:**
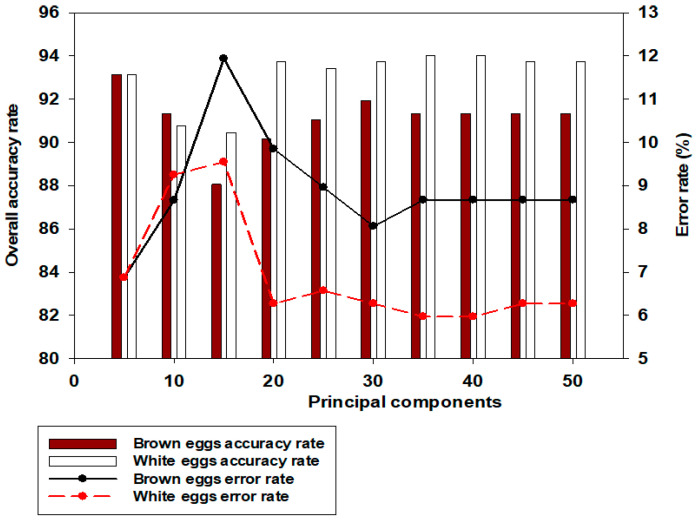
Model accuracies and error rates for all PCs from 5 to 50 on day 0 of incubation (see Table A2).

Figure 5 shows model accuracies and error rates on day 0 of incubation, for all PCs considered from 5 to 50. It was observed that using PCs above 5 poses a risk to the model’s robustness, as the misclassification error rates are found to increase after 5 PCs. Table 3 shows typical day 0 incubation confusion matrix results at some other selected thresholds 0.81 and 0.55, for both brown and white eggs. Detailed values used for the computation of the confusion matrices is as shown in Appendix A. On day 0 of incubation for brown eggs (Table 3c), TPR of 100% was achieved at a threshold value of 0.81, whereas white eggs achieved a TPR classification accuracy of 95.24% at this same threshold (Table 3d). Detailed percent classification accuracy information for both brown and white eggs are shown in Table A1 and Table A2, for 25 PCs. Similar detailed percent classification accuracies for 5PCs are shown in the Supplementary Materials. The same TPR classification accuracy of 95.24% was also achieved at a TR of 0.80 for white eggs, in which only one non-fertile egg was misclassified as fertile (Table A2).

None of the threshold values considered between 0.50 and 0.85 for the white eggs on day 0 of incubation achieved 100% classification accuracy, considering the TPR values. Considering the true negative rates (TNR), however, white eggs achieved an accuracy of 100% at four threshold values of 0.5, 0.55, 0.60, and 0.65, whereas brown eggs achieved 99.68, 99.68, 99.68, and 99.04%, respectively, at these same thresholds (Table A1, Table A2 and Table 3a,b). For the brown eggs, only one fertile egg was misclassified as non-fertile at thresholds 0.5, 0.55, and 0.60, but three fertile eggs were misclassified as non-fertile at threshold value of 0.65. These results showed that the PLS algorithm used can discriminate both brown and white fertile eggs from non-fertile eggs prior to incubation, using any of the thresholds identified. This is a huge contribution when compared to earlier studies that have not achieved such accuracies prior to incubation, with some studies reporting only the overall accuracy metric, which cannot capture the prevalent class overfitting problem in a rare-class data scenario [17,18,19,20,33]. Emerging studies should deliberately consider handling the imbalanced data occurrence and feasibility of building a multi-purpose singular model to handle both brown and white eggs.

The results shown specifically in Table 3a–d at 25 PCs are promising for both brown and white eggs, considering the closer margin of TPR and TNR accuracies. However, models built with 5 PCs as shown in Table 3e–h are much in favor of the prevalent class, as can be seen in the perfect TNR accuracies against the lowest TPR accuracies. This observation has been reported in the literature to be related to the imbalanced data phenomenon [34,40,41] in the considered data sets. Therefore, despite how the overall percentage accuracy (OVA) obtained for brown eggs on day 0 of incubation at various thresholds from 0.50 to 0.75 at 25 PCs were much higher than that at the thresholds of 0.80 and 0.81 (see Table A1), the final accepted model might not be based on this overall accuracy due to the imbalanced data phenomenon, shifting the overall accuracy performance in favor of the majority class at the expense of the minority class. This is the reason why performance is better judged based on the true positive and/or true negative rates. In the specific situation under discussion, it might be more appropriate to adopt a model based on the 0.81 TR (TPR value of 100%, OVA of 91.04%), than a model based on 0.55 TR (TPR value of 86.96% but OVA of 98.81%). Notwithstanding, if the majority class is also of equal or greater interest, the reverse choice might be preferable, in which a model based on the 0.55 TR (TNR 99.68%, OVA 98.81%) would be adopted over that based on TR 0.81 (TNR 90.38%, OVA 91.04). Also see Table 3a,c. Our study has clearly shown that the adapted PLS regression algorithm is adequate for handling chicken egg classification task, especially on day 0 of incubation. There is, however, a need to improve its present implementation mode, in relation to handling imbalanced data, towards achieving a better trade-off between TPR and TNR accuracies, and, at the same time, favoring the use of a fewer PLS components. Likewise, future work should extend to considering the feasibility of building a single multi-purpose model to handle chicken egg fertility classification, irrespective of the eggshell pigmentation.

## 4. Conclusions

This paper has presented the details of a study carried out to investigate the appropriateness of a PLS regression-based technique to classify chicken egg fertility data. While 25 PCs were found to be suitable for accurate classification based on the true positive rate computation, only 5 PCs proved to be appropriate using the true negative and misclassification error rate computations. The analysis results showed that the adapted PLS regression algorithm can discriminate both brown and white fertile eggs from non-fertile eggs prior to incubation and on different days of incubation using the moving-thresholding selection technique. It was further observed that recognizing appropriately fertile eggs (TNR of 100%) with an acceptable matching up recognition accuracy of non-fertile eggs would need up to 25 PCs.

Models built with 5 PCs on the other hand, shifted recognition accuracies to be mostly in favor of the majority of fertile egg classes at the expense of the rare class non-fertile eggs. This scenario has been attested to being related to the imbalanced data problem. We, therefore, need a classifier mode that will perform at a much closer margin of TPR and TNR accuracies and, at the same time, use much fewer PCs. This study has clearly shown that the simple PLS regression algorithm is adequate for handling the chicken egg classification task, prior to incubation. There is, however, a need to improve its present implementation mode, in relation to handling imbalanced data, towards achieving a better trade-off between TPR and TNR accuracies, and, at the same time, optimizing the use of an adequate number of PCs. Future work will also address the imbalance data problem and, likewise, consider the feasibility of building a single multi-purpose model to handle chicken egg fertility classification, irrespective of the eggshell pigmentation.

## Figures and Tables

**Table 1:** Overall egg sample specifications for (a), brown eggs and (b), white eggs.

**a.**					
**Incubation Period**	**Egg Received**	**Broken**	**Total Eggs Used**	**Fertile (F)**	**Non-Fertile (NF)**
Day 0	336	1	335	312 (93.13%)	23 (6.87%)
Day 1	335	1	334	311 (93.11%)	23 (6.89%)
Day 2	334	-	334	311 (93.11%)	23 (6.89%)
Day 3	334	-	334	311 (93.11%)	23 (6.89%)
Day 4	334	-	334	311 (93.11%)	23 (6.89%)
**b.**					
**Incubation Period**	**Egg Received**	**Broken**	**Total Eggs Used**	**Fertile (F)**	**Non-Fertile (NF)**
Day 0	336	1	335	314 (93.73%)	21 (6.27%)
Day 1	335	-	335	314 (93.73%)	21 (6.27%)
Day 2	335	-	335	314 (93.73%)	21 (6.27%)
Day 3	335	2	333	312 (93.69%)	21 (6.31%)
Day 4	333	-	333	312 (93.69%)	21 (6.31%)

**Table 2:** Confusion matrix.

		Prediction Class	
		Predicted as Positive	Predicted as Negative
**True class**	Actually Positive	TPR	FNR
	Actually Negative	FPR	TNR

**Table 3:** Typical confusion matrix for selected egg models at different thresholds and PCs (a) brown, TR 0.55, PC 25; (b) white, TR 0.55, PC 25; (c) brown, TR 0.81, PC 25; (d) white, TR 0.81, PC 25; (e) brown, TR 0.55, PC 5; (f) white, TR 0.55, PC 5; (g) brown, TR 0.81, PC 5; and (h) white, TR 0.81, PC 5.

(a)	**Prediction Class (%)**		
		Predicted	Predicted
**True Class (%)**		Positive	Negative
	Actually Positive	86.96 (20/23)	13.04 (3/23)
	Actually Negative	0.32 (1/312)	99.68 (311/312)
OVA = 98.81%			
(b)	**Prediction class (%)**		
		Predicted	Predicted
**True Class (%)**		Positive	Negative
	Actually Positive	61.90 (13/21)	38.10 (8/21)
	Actually Negative	0.00 (0/312)	100.00 (314/314)
OVA = 97.61%			
(c)	**Prediction class (%)**		
		Predicted	Predicted
**True Class (%)**		Positive	Negative
	Actually Positive	100.00 (23/23)	0.00 (0/23)
	Actually Negative	9.62 (30/312)	90.38 (282/312)
OVA = 91.04%			
(d)	**Prediction class (%)**		
		Predicted	Predicted
**True Class (%)**		Positive	Negative
	Actually Positive	95.24 (20/21)	4.76 (1/21)
	Actually Negative	7.32 (23/314)	92.68 (291/314)
OVA = 92.84%			
(e)	**Prediction class (%)**		
		Predicted	Predicted
**True Class (%)**		Positive	Negative
	Actually Positive	0.00 (0/23)	100.00 (23/23)
	Actually Negative	0.00 (0/312)	100.00 (312/312)
OVA = 93.13%			
(f)	**Prediction class (%)**		
		Predicted	Predicted
**True Class (%)**		Positive	Negative
	Actually Positive	0.00 (0/21)	100.00 (21/21)
	Actually Negative	0.00 (0/314)	100.00 (314/314)
OVA = 93.73%			
(g)	**Prediction class (%)**		
		Predicted	Predicted
**True Class (%)**		Positive	Negative
	Actually Positive	0.00 (0/23)	100.00 (23/23)
	Actually Negative	0.00 (0/312)	100.00 (312/312)
OVA = 93.13%			
(h)	**Prediction class (%)**		
		Predicted	Predicted
**True Class (%)**		Positive	Negative
	Actually Positive	4.76 (1/21)	95.24 (20/21)
	Actually Negative	0.00 (0/314)	98.73 (310/314)
OVA = 92.84%			

## Data Availability

Data are contained within the article and Supplementary Materials.

## Supplementary Materials

The following supporting information can be downloaded at: https://www.mdpi.com/article/10.3390/s24051485/s1. Table S1: Percent classification accuracy for brown eggs based on 5 PLS components. Table S2: Percent classification accuracy for white eggs based on 5 PLS components.

## Appendix A

**Table A1:** Percent classification accuracy for brown eggs based on 25 PLS components.

INC. Day	TR	FP	FN	TP	TN	TPR (%)	TNR (%)	OVA (%)
Day 0	0.5	1	8	15	311	65.22	99.68	97.31
F = 312	0.55	1	3	20	311	86.96	99.68	98.81
NF = 23	0.60	1	3	20	311	86.96	99.68	98.81
T = 335	0.65	3	3	20	309	86.96	99.04	98.21
	0.70	12	3	20	300	86.96	96.15	95.52
	0.75	14	1	22	298	95.65	95.51	95.52
	0.80	29	1	22	283	95.65	90.71	91.04
	0.81	30	0	23	282	100	90.38	91.04
Day 1	0.5	0	10	13	311	56.52	100	97.01
F = 311	0.55	0	8	15	311	65.22	100	97.6
NF = 23	0.60	1	4	19	310	82.61	99.68	98.5
T = 334	0.65	3	2	21	308	91.3	99.04	98.5
	0.70	6	1	22	305	95.65	98.07	97.9
	0.75	16	1	22	295	95.65	94.86	94.91
	0.79	32	1	22	279	95.65	89.71	90.12
	0.80	37	0	23	274	100	88.1	88.92
Day 2	0.5	0	4	19	311	82.61	100	98.8
F = 311	0.55	0	3	20	311	86.96	100	99.1
NF = 23	0.60	0	1	22	311	95.65	100	99.7
T = 334	0.65	0	1	22	311	95.65	100	99.7
	0.70	2	1	22	309	95.65	99.36	99.1
	0.75	8	1	22	303	95.65	97.43	97.31
	0.79	18	1	22	293	95.65	94.21	94.31
	0.80	21	1	22	290	95.65	93.25	93.41
Day 3	0.5	0	7	16	311	69.57	100	97.9
F = 311	0.55	0	7	16	311	69.57	100	97.9
NF = 23	0.60	0	6	17	311	73.91	100	98.2
T = 334	0.65	1	4	19	310	82.61	99.68	98.5
	0.70	5	3	20	306	86.96	98.39	97.6
	0.75	15	2	21	296	91.3	95.18	94.91
	0.79	25	0	23	286	100	91.96	92.51
	0.80	28	0	23	283	100	91	91.62
Day 4	0.5	0	6	17	311	73.91	100	98.2
F = 311	0.55	0	5	18	311	78.26	100	98.5
NF = 23	0.60	0	5	18	311	78.26	100	98.5
T = 334	0.65	1	3	20	310	86.96	99.68	98.8
	0.70	4	0	23	307	100	98.71	98.8
	0.75	13	0	23	298	100	95.82	96.11
	0.79	17	0	23	294	100	94.53	94.91
	0.80	20	0	23	291	100	93.57	94.01

**Table A2:** Percent classification accuracy for white eggs based on 25 PLS components.

INC. Day	TR	FP	FN	TP	TN	TPR (%)	TNR (%)	OVA (%)
Day 0	0.5	0	10	11	314	52.38	100	97.01
F = 314	0.55	0	8	13	314	61.9	100	97.61
NF = 21	0.60	0	7	14	314	66.67	100	97.91
T = 335	0.65	0	6	15	314	71.43	100	98.21
	0.70	2	3	18	312	85.71	99.36	98.51
	0.75	7	3	18	307	85.71	97.77	97.01
	0.80	21	1	20	293	95.24	93.31	93.43
	0.81	23	1	20	291	95.24	92.68	92.84
Day 1	0.5	0	5	16	314	76.19	100	98.51
F = 314	0.55	0	3	18	314	85.71	100	99.1
NF = 21	0.60	1	3	18	313	85.71	99.68	98.81
T = 335	0.65	2	2	19	312	90.48	99.36	98.81
	0.70	6	2	19	308	90.48	98.09	97.61
	0.75	10	1	20	304	95.24	96.82	96.72
	0.80	21	1	20	293	95.24	93.31	93.43
	0.81	26	1	20	288	95.24	91.72	91.94
Day 2	0.5	0	8	13	314	61.9	100	97.61
F = 314	0.55	0	5	16	314	76.19	100	98.51
NF = 21	0.60	0	3	18	314	85.71	100	99.1
T = 335	0.65	2	1	20	312	95.24	99.36	99.1
	0.70	3	1	20	311	95.24	99.04	98.81
	0.75	10	1	20	304	95.24	96.82	96.72
	0.80	26	0	21	288	100	91.72	92.24
	0.81	31	0	21	283	100	90.13	90.75
Day 3	0.5	0	4	17	312	80.95	100	98.8
F = 312	0.55	0	3	18	312	85.71	100	99.1
NF = 21	0.60	0	3	18	312	85.71	100	99.1
T = 333	0.65	1	2	19	311	90.48	99.68	99.1
	0.70	3	2	19	309	90.48	99.04	98.5
	0.75	10	1	20	302	95.24	96.79	96.7
	0.79	23	0	21	289	100	92.63	93.09
	0.80	27	0	21	285	100	91.35	91.89
Day 4	0.5	0	5	16	312	76.19	100	98.5
F = 312	0.55	0	5	16	312	76.19	100	98.5
NF = 21	0.60	1	3	18	311	85.71	99.68	98.8
T = 333	0.65	2	0	21	310	100	99.36	99.4
	0.70	3	0	21	309	100	99.04	99.1
	0.75	5	0	21	307	100	98.4	98.5
	0.79	12	0	21	300	100	96.15	96.4
	0.80	13	0	21	299	100	95.83	96.1
