# Supplementary material for: An Adaptive Partial Least-Squares Regression Approach for Classifying Chicken Egg Fertility by Hyperspectral Imaging
Source: Sensors (Basel). 2024 Feb 24;24(5):1485. doi: 10.3390/s24051485 (PMC10934929; doi:10.3390/s24051485)
Supplement: Supplementary file 1 [file sensors-24-01485-s001.zip › sensors-2576310-supplementary.pdf]

Table S1. Percent classification accuracy for brown eggs based on 5 PLS components.

INC. DAY	THRESH.	FP	FN	TP	TN	TPR (%)	TNR (%)	OVA (%)
**Day 0**	0.5	0	23	0	312	0	100	93.13
F = 312	0.55	0	23	0	312	0	100	93.13
NF = 23	0.60	0	23	0	312	0	100	93.13
T = 335	0.65	0	23	0	312	0	100	93.13
	0.70	0	23	0	312	0	100	93.13
	0.75	0	23	0	312	0	100	93.13
	0.80	0	23	0	312	0	100	93.13
	0.81	0	23	0	312	0	100	93.13
**Day 1**	0.5	0	23	0	311	0	100	93.11
F = 311	0.55	0	23	0	311	0	100	93.11
NF = 23	0.60	0	23	0	311	0	100	93.11
T = 334	0.65	0	23	0	311	0	100	93.11
	0.70	0	23	0	311	0	100	93.11
	0.75	0	23	0	311	0	100	93.11
	0.79	0	23	0	311	0	100	93.11
	0.80	0	23	0	311	0	100	93.11
**Day 2**	0.5	0	23	0	311	0	100	93.11
F = 311	0.55	0	23	0	311	0	100	93.11
NF = 23	0.60	0	23	0	311	0	100	93.11
T = 334	0.65	0	23	0	311	0	100	93.11
	0.70	0	23	0	311	0	100	93.11
	0.75	0	22	1	311	4.35	100	93.41
	0.79	1	22	1	310	4.35	99.68	93.11
	0.80	1	22	1	310	4.35	99.68	93.11
**Day 3**	0.5	0	23	0	311	0	100	93.11
F = 311	0.55	0	22	1	311	4.35	100	93.41
NF = 23	0.60	1	22	1	310	4.35	99.68	93.11
T = 334	0.65	1	22	1	310	4.35	99.68	93.11
	0.70	1	22	1	310	4.35	99.68	93.11
	0.75	1	20	3	310	13.04	99.68	93.71
	0.79	4	19	4	307	17.39	98.71	93.11
	0.80	4	19	4	307	17.39	98.71	93.11
**Day 4**	0.5	0	20	3	311	13.04	100	94.01
F = 311	0.55	1	19	4	310	17.39	99.68	94.01
NF = 23	0.60	1	18	5	310	21.74	99.68	94.31
T = 334	0.65	1	17	6	310	26.09	99.68	94.61
	0.70	2	17	6	309	26.09	99.36	94.31
	0.75	2	15	8	309	34.78	99.36	94.91
	0.79	5	15	8	306	34.78	98.39	94.01
	0.80	8	14	9	303	39.13	97.43	93.41

Table S2. Percent classification accuracy for white eggs based on 5 PLS components.

INC. DAY	THRESH.	FP	FN	TP	TN	TPR (%)	TNR (%)	OVA (%)
**Day 0**	0.5	0	21	0	314	0	100	93.73
F = 314	0.55	0	21	0	314	0	100	93.73
NF = 21	0.60	0	21	0	314	0	100	93.73
T = 335	0.65	0	21	0	314	0	100	93.73
	0.70	0	21	0	314	0	100	93.73
	0.75	0	21	0	314	0	100	93.73
	0.80	3	20	1	311	4.76	99.04	93.13
	0.81	4	20	1	310	4.76	98.73	92.84
**Day 1**	0.5	0	21	0	314	0	100	93.73
F = 314	0.55	0	21	0	314	0	100	93.73
NF = 21	0.60	0	21	0	314	0	100	93.73
T = 335	0.65	0	21	0	314	0	100	93.73
	0.70	0	21	0	314	0	100	93.73
	0.75	0	21	0	314	0	100	93.73
	0.80	1	21	0	313	0	99.68	93.43
	0.82	3	21	0	311	0	99.04	92.84
**Day 2**	0.5	0	21	0	314	0	100	93.73
F = 314	0.55	0	21	0	314	0	100	93.73
NF = 21	0.60	0	21	0	314	0	100	93.73
T = 335	0.65	0	21	0	314	0	100	93.73
	0.70	0	21	0	314	0	100	93.73
	0.75	0	21	0	314	0	100	93.73
	0.80	1	20	1	313	4.76	99.68	93.73
	0.82	5	18	3	309	14.29	98.41	93.13
**Day 3**	0.5	0	21	0	312	0	100	93.69
F = 312	0.55	0	21	0	312	0	100	93.69
NF = 21	0.60	0	21	0	312	0	100	93.69
T = 333	0.65	0	21	0	312	0	100	93.69
	0.70	1	20	1	311	4.76	99.68	93.69
	0.75	2	19	2	310	9.52	99.36	93.69
	0.79	7	18	3	305	14.29	97.76	92.49
	0.80	8	18	3	304	14.29	97.44	92.19
**Day 4**	0.5	1	14	7	311	33.33	99.68	95.50
F = 312	0.55	1	14	7	311	33.33	99.68	95.50
NF = 21	0.60	1	13	8	311	38.10	99.68	95.80
T = 333	0.65	4	10	11	308	52.38	98.72	95.80
	0.70	5	10	11	307	52.38	98.40	95.50
	0.75	8	7	14	304	66.67	97.44	95.50
	0.79	10	7	14	302	66.67	96.79	94.89
	0.80	12	7	14	300	66.67	96.15	94.29
